# Predictors of trajectories of obsessive-compulsive symptoms during the COVID-19 pandemic in the general population in Germany

**DOI:** 10.1038/s41398-021-01419-2

**Published:** 2021-05-27

**Authors:** Lena Jelinek, Anja S. Göritz, Franziska Miegel, Steffen Moritz, Levente Kriston

**Affiliations:** 1 Department of Psychiatry and Psychotherapy, University Medical Center Hamburg-Eppendorf, Hamburg, Germany; 2 Occupational and Consumer Psychology, University of Freiburg, Freiburg im Breisgau, Germany; 3 Department of Medical Psychology, University Medical Center Hamburg-Eppendorf, Hamburg, Germany

**Keywords:** Pathogenesis, Psychology

## Abstract

The COVID-19 pandemic has been associated with an increase in obsessive-compulsive disorder/symptoms (OCD/OCS). However, knowledge is limited regarding the trajectories of OCS during the pandemic, as well as their predictors and mechanisms (e.g., experiential avoidance, EA). The aim of this study was to describe the trajectories of OCS and the identification of associated factors. We assessed 1207 participants of the general population in March 2020 (t1) and June 2020 (t2). Pre-pandemic data was available from March 2014 for a subsample (*n* = 519). To define trajectories, we determined OCS status (OCS+/−). We performed a hierarchical multinomial logistic regression to investigate predictors of trajectories. Between t1 and t2, 66% of participants had an asymptomatic trajectory (OCS−/OCS−); 18% had a continuously symptomatic trajectory (OCS+/OCS+). Ten percent had a delayed-onset trajectory (OCS−/OCS+), and the recovery trajectory group (OCS+/OCS−) was the smallest group (6%). Higher education reduced the odds of an OCS+/OCS− trajectory. OCS in 2014 was associated with increased odds of showing an OCS+/OCS+ or OCS−/OCS+ trajectory. When EA at t1 and change in EA from t1 to t2 were added to the model, higher EA at t1 was associated with increased odds of scoring above the cut score on one or more of the assessments. A higher decrease in EA from t1 to t2 reduced the probability of showing an OCS+/OCS+ and an OCS−/OCS+ trajectory. While the current data supports a slight increase in OCS during the pandemic, trajectories differed, and EA seems to represent an important predictor for an unfavorable development.

## Introduction

The worldwide lifetime prevalence for obsessive-compulsive disorder (OCD) has been estimated at 2–3%^1,2^. Subclinical obsessive-compulsive symptoms (OCS), however, are more common and are experienced by over 20% of the general population^1,3^. Approximately 56% of people with OCD experience obsessions (i.e., recurrent and persistent thoughts) related to contamination (C-OCD/C-OCS)^4^, for example, the fear of getting a serious disease oneself or of contaminating others with a virus (e.g., human immunodeficiency virus/ HIV) or a bacterial infection (e.g., enterohemorrhagic *Escherichia coli*/EHEC). During the COVID-19 pandemic, fear of contamination has become prevalent worldwide, and ritualized washing behaviors have not only become standard but are advocated by organizations such as the World Health Organization (WHO). Similarly, avoidance behavior has been mandated by governments in the form of lockdowns and official instructions for social distancing. Hence, thoughts and behaviors that are routinely held and carried out by people with C-OCS have suddenly been experienced by people with low OCS. This may potentially lead to an increase in OCS in some people and increase the incidence of OCD^5^. Moreover, pandemic-related events may include stressful life events for some people (e.g., the sudden death of a loved one) that may increase OCS/OCD, as indicated by recent two-year twin data^6^. Moreover, an exacerbation of OCS during the Coronavirus SARS-CoV-2 (COVID-19) pandemic has been suggested in people with manifest OCD, especially people with C-OCD/C-OCS, at the start of the pandemic^7,8^.

To date, the body of evidence is conflicting. Some studies suggest an increase in OCS in people with manifest OCD during the pandemic^9–13^, whereas others do not^14^. In nonclinical samples, evidence has been reported for an increase in OCS^15–18^. However, most investigations suffer from using a cross-sectional design and only the recent study by Cox and Olantunji^18^ was able to draw upon data from 2016 reporting an increase with a small effect size (*d* = 0.1) from 2016 to April 2020. While on average OCS may increase, the trajectories of symptomatology during the COVID-19 pandemic are manifold, and data on the trajectories in the general population are currently lacking.

Furthermore, predictors and process candidates associated with different trajectories of OCS are of interest as they may offer insight on people at risk in future pandemics and mechanisms of change important for treatment. Three months after the quarantine was lifted in Wuhan, the presence of OCD was associated with being single, a student, as well as a family history of mental disorders, psychiatric comorbidity, and longer sleep latency^16^. Diagnosis of OCD, however, relied on a cut-off score of 6 for the self-rating version of the Yale-Brown Obsessive-Compulsive Scale (Y-BOCS). While the self-rating version has shown good convergence with the clinician version, a cut-off score of 16 has been established^19^. In the study by Abba-aji et al.^15^, worry about germs and ritualized cleaning behavior (which may largely overlap with C-OCS) was, for example, associated with male gender, having post-secondary education, and age >60 years. However, these findings are limited by the cross-sectional design and the methods of assessing OCS (i.e., retrospective assessment of pre-pandemic OCS, the assessment of a selective help-seeking sample, and the reliance on an ad hoc measure). Further, pre-pandemic OCS and insomnia symptoms have been suggested to be predictive of OCS^18^. From data collected during other disease outbreaks (such as influenza A (H1N1)/swine flu, Zika virus), we know that anxiety regarding a specific virus is predicted by obsessive-compulsive beliefs and OCS, health anxiety, and contamination fears, as well as disgust sensitivity (influenza A (H1N1)/swine flu^20,21^) and that contamination severity, is overestimated (Zika virus^22^, Ebola^23^). However, in these studies fear of a specific disease was predicted, not OCS/OCD.

So far two attempts both using a cross-sectional design to investigate processes involved in the formation of OCD/OCS during a pandemic were made^24,25^. Wheaton et al.^25^ assessed a US Amazon MTurk sample of 738 adults and were able to show that intolerance of uncertainty, a factor assumed to be involved in the etiology of OCD, partially mediated the relation between COVID-19 related concerns and OCD. Seçer and Ulaş^24^ assessed 598 high school students in Turkey and reported that the relationship between fear of COVID-19 and OCS was mediated by emotional reactivity, experiential avoidance (EA), and depression-anxiety. EA (i.e. psychological inflexibility, see refs. ^26,27^) has been suggested to represent a core mechanism in the etiology of many psychological problems and disorders, e.g., Kashdan et al.^28^. It refers to the “excessive negative evaluations of unwanted private thoughts, feelings, and sensations, an unwillingness to experience these private events, and deliberate efforts to control or escape from them” ^28^^, p. 1301^. As such, EA is thought to explain the difference between unwanted intrusive thoughts experienced by the majority of the population, (e.g., Radomsky et al.^29^), and obsessions as part of OCD^26^. Accordingly, it has also been shown to represent a mechanism of change within the treatment of OCD/OCS^30^. Although Seçer and Ulaş^24^ underline the relevance of EA during the COVID-19 pandemic, their cross-sectional design does not allow for a causal interpretation.

To summarize, OCS may manifest at different time points during a pandemic and take different courses. Moreover, factors that predict the changes in OCS are not well understood. Preliminary data show that an increase in OCS during the COVID-19 pandemic is associated with male gender, post-secondary education, age >60 years^15^, and pre-pandemic C-OCS^9^, as well as OCS^18^. Naturalistic, longitudinal data have emphasized the role of major depression in the chronic course of OCD^31^. Moreover, EA has been shown to mediate the relation between fear of COVID-19 and OCS in high school students^24^. The aims of this study were twofold. First, we wanted to describe the frequency of different OCS trajectories during the COVID-19 pandemic in the general population. Second, we sought to investigate factors that have predicted an unfavorable development, such as demographic (gender, age, education) and psychopathological factors (C-OCS, comorbid depression, EA). Due to the preliminary evidence and the lack of data in Germany on OCS during the COVID-19 pandemic at the time the study was conducted, we did not have firm hypotheses at the time the study was set up. Still, we expected a general increase in OCS over the first three months (March to June 2020) of the pandemic in Germany. Moreover, we assumed that a positive screening for OCS (score above the German cut-off on the Obsessive-Compulsive Inventory-Revised, OCI-R) on one or both of the assessments (in March and June 2020) would be associated with male gender, greater age, higher level of education, and higher pre-pandemic levels of depression and pre-pandemic C-OCS, as well as higher levels of EA at the start of the pandemic.

The results of this study may help to predict OCS trajectories as well as people at risk in future pandemics and inform interventions that target OCD.

## Methods

### Recruitment and procedure

Participants were recruited via WisoPanel® at www.wisopanel.net, which is a service providing scientists with the opportunity to recruit a large number of participants from the general population (for further information on WisoPanel,^32–35^). Assessments were performed at three points in time using the online platform Unipark/Questback® (Globalpark AG). In 2020, participants were assessed between March 21 and March 30 (t1) and were reassessed three months later between June 22 and June 30 (t2). During t1, a COVID-19-induced lockdown (e.g., restricted social contact) in Germany had just been announced. Assessment at t2 was performed soon after the easing of lockdown restrictions (e.g., opening of restaurants). For baseline data (t0), we were able to draw on data for a subsample of the participants. These were part of a larger study (*N* = 2048; *n* = 1203 [58.7%] female; age: *M* = 52.79 [*SD* = 14.30]; *n* = 1287 [62.8%] with A levels; *n* = 366 [17.9%] with OCS according to the German OCI-R cut-off) that was assessed between March 30 and April 7, 2014. For a previous investigation of this sample see Moritz et al.^36^.

The study was conducted in accordance with the Declaration of Helsinki and was approved by the ethics committee (#LPEK-0129). As a reward for participation, participants received a link to download a PDF manual on techniques to improve self-esteem (t1) and a PDF manual on cognitive-behavioral strategies to improve mental health during the COVID-19 pandemic (t2).

### Assessment

The German version of the Obsessive-Compulsive Inventory-Revised (OCI-R^37,38^) was used to assess OCS. Symptom dimensions are assessed by six subscales: washing, obsessing, checking, neutralizing, hoarding, and ordering. For the English version, clinical benchmarks and norm value exist^39^. Test-retest reliability of *r*_tt_ = 0.82 for a clinical OCD sample and *r*_tt_ = 0.84 for a nonclinical sample has been reported^40^. Moreover, OCI-R has shown to be sensitive to change^41^, and equivalent results were reported for the paper-and-pencil and web-based administration^42^. Good psychometric properties have been shown for the German version of the OCI-R^40,43^. In this study, internal consistency for the OCI-R was Cronbach’s *α* = 0.92. A cut-off score of 18 for the total score and of 3 for the washing subscale has been suggested for the German version of the OCI-R^44^ and a cut-off score of 21 for the original English version^39^.

At t0, the Patient Health Questionnaire (PHQ-9^45,46^) was used to assess depressive symptoms. The validity and reliability of the German version are good^46,47^. Internal consistency for the PHQ-9 was good (Cronbach’s *α* = 0.91) in this study.

The Acceptance and Action Questionnaire for Obsessions and Compulsions (AAQ-OCD^26^) assesses EA specific to obsessions and compulsions based on the Acceptance and Action Questionnaire-II^48^. The AAQ-OCD was adapted for this study to the current COVID-19 pandemic (AAQ-OCD-COVID) to assess participants’ experiential avoidance of obsessions and compulsions that were related to the COVID-19 pandemic at t1 and t2 (item example: “My intrusive thoughts related to the COVID-19 pandemic determine the actions that I take”). Items were rated on a 7-point Likert scale ranging from 1 (*never true*) to 7 (*always true*). Psychometric properties of the AAC-OCD have been reported to be good^26^. In this study, internal consistency for the AAQ-OCD-COVID was good (Cronbach’s *α* = 0.93).

### Data analysis

To define distinct trajectory groups, OCS status (OCS+/–) was determined for t1 and t2 using the clinical cut-off score of ≥18 for the German OCI-R, which led to four potential trajectories: (1) the *continuously symptomatic trajectory* with OCS at t1 and t2 (OCS+/OCS+), (2) the *recovery group trajectory* with OCS at t1 but not at t2 (OCS+/OCS−), (3) the *delayed-onset trajectory* without OCS at t1 but at t2 (OCS−/OCS+), and (4) the *asymptomatic trajectory* (OCS−/OCS−). Results were recalculated for the English cut-off score of 21 (sensitivity analyses).

We performed hierarchical multinomial regression to examine which factors were associated with group membership using OCS−/OCS− as the reference category. First, we investigated variables that might influence OCS trajectory membership. These included sociodemographics (age, gender, education), depressive symptoms (PHQ-9) at t0, OCS at t0 (OCS+/–), and phenotype of the OCS at t0 (contamination [C+] vs. [C−] other as determined by the cut-off of the washing subscale ≥3 vs. <3, C+/–). As OCS+/– and contamination focus at t0 (C+/–) were assessed by the same measure (OCI-R) and were very highly correlated, we combined them into a single variable with three categories: participants with *pre-pandemic contamination-related OCS* (C-OCS, i.e., OCS+ and C+), participants with *pre-pandemic contamination-unrelated OCS* (nC-OCS; i.e., OCS+ and C–), and *participants without pre-pandemic OCS* (OCS−). In a second step, EA at t1 and change in EA from t1 to t2 were added to the model to assess whether EA had additional explanatory value over the t0 variables and thus might be considered a potential proximal process variable. To check for collinearity, we calculated the correlation between EA at t1 and change in EA. Although the association was substantial (0.48) the unique variance of the variables was still high enough (above 75%) to allow estimation of their unique effects.

Our primary analysis included all cases (i.e., it also included cases with missing data on one or more predictors) and used all available information in the model to account for missing data by applying robust full information maximum-likelihood estimation in MPlus 7.11^49^. To test the robustness of the findings, sensitivity analyses were carried out that included only those participants with complete data on predictors in SPSS 25.

Besides the analyses using the trajectory groups, we calculated change in OCS using the full scale of the OCI-R and its subscale with repeated measure analyses of variances. We also calculated multiple hierarchical linear regression models with change in OCS over the pandemic (OCI-R total scores at t1 minus OCI-R total scores at t2) entered as the dependent variable. As in the multinomial regression, demographic background variables (age, gender, education), depressive symptoms at t0 (PHQ-9 total score), OCS at t0 (C-OCS, nC-OCS, OCS−) were entered as predictors in the first block. EA (AAQ-OCD-COVID) at t1 and change in EA from t1 to t2 were entered as the second block.

As a measure of effect size, Cohen’s *d* was calculated (according to Lenhard and Lenhard^50^), applying Cohen’s rules of thumb for evaluation with *d* ≈ 0.2, ≈ 0.5, and ≈ 0.8, corresponding to small, medium, and large effects^51^. Findings with *p* < 0.05 were considered statistically significant. In order to account for a large number of statistical hypothesis tests in the study, we also report which findings were statistically significant after applying the Holm-Bonferroni procedure to prevent the family-wise error rate in the main analyses from exceeding 0.05^52^.

## Results

### Sample

A total of *N* = 14285 individuals from the general population were invited to participate at t1. Of these, 2287 (16%) completed the OCI-R (*n* = 2727 [19%] accessed the survey). On average, the responders were older (*M* = 52.78, *SD* = 14.39) than the nonresponders (*M* = 46.95, *SD* = 14.17, *t*[14283] = 5.830, *p* < 0.001, *d* = 0.410), more likely to be male (42.3% vs. 38.6%; *χ*^2^[1] = 12.317, *p* < 0.001, *d* = 0.058), and more likely to have an A-level degree (61.3% vs. 55.1%, *χ*^2^[1] = 34.343, *p* < 0.001, *d* = 0.098). Of the participants completing the OCI-R, we excluded *n* = 32 (1.4%), that is, participants who indicated in the final question of the survey that they had answered the questions of the survey untruthfully (*n* = 6) or those who exhibited a stereotypical answer pattern (i.e., same value apart from 0) in their OCI-R ratings (*n* = 26). Thus, the sample at t1 comprised *n* = 2255 participants with a mean age of 53 years (*M* = 53.36 years, *SD* = 14.25), including *n* = 1397 women (58.0%), and *n* = 1381 with an A level degree (61.3%). Of these, *n* = 1207 (53.5%) completed the OCI-R at t2, representing the final sample (for demographics, see Table 1). Sociodemographics and clinical variables of the final sample (with available data at t1 and t2) were largely comparable (Cohen’s *d* ≤ 0.2) to participants with data only available for t1 (age: *t*[2164.02] = 8.928, *p* < 0.001, *d* = 0.177; gender: *χ*^2^[1] = 8.748, *p* = 0.003, *d* = 0.098; educational level: *χ*^2^[1] = 19.171, *p* < 0.001, *d* = 0.146; OCI-R at t1: *t*[2250.31] = 0.452, *p* = 0.651, *d* = 0.02; AAQ-OCD-COVID at t1: *t*[2189] = 0.239, *p* = 0.811, *d* = 0.01) and to participants with available data for all three-time points (*n* = 519; age: *t*[1155.11] = 3.551, *p* < 0.001, *d* = 0.204; gender: *χ*^2^[1] = 0.213, *p* = 0.645, *d* = 0.041; educational level: *χ*^2^[1] = 1.086, *p* = 0.297, *d* = 0.092, OCI-R at t1: *t*[1205] = 2.229, *p* = 0.026, *d* = 0.13; AAQ-OCD-COVID at t1: *t*[1162.96] = 0.975, *p* = 0.330, *d* = 0.06).

**Table 1:** Demographics by OCS trajectory group: means and standard deviations (number and percent).

	OCS+/OCS+ (+/+)			OCS−/OCS+ (−/+)			OCS+/OCS− (+/−)			OCS−/OCS− (−/−)			Total sample			Test of difference between groups^a^
	*n*	*M/n*	*SD*/*%*	*n*	*M/n*	*SD*/*%*	*n*	*M/n*	*SD*/*%*	*n*	*M/n*	*SD*/*%*	*n*	*M/n*	*SD*/*%*	
Age, t1	216	53.88	14.25	120	56.83	14.55	71	54.56	13.82	800	56.32	13.22	1207	55.83	13.60	*F*(3, 1203) = 2.259, *p* = 0.080
Sex (women)	216	113	52.31	120	72	60.00	71	33	46.48	800	447	55.88	1207	665	55.10	*χ*^2^ (3) = 4.169, *p* = 0.244
Education (≥A levels), t1	216	114	52.78	120	65	54.17	71	37	52.11	800	474	59.25	1207	517	42.83	*χ*^2^ (3) = 4.299, *p* = 0.231
COVID-19 risk group, t1	214	112	52.34	118	69	58.47	69	30	43.48	786	420	53.44	1186	633	53.37	*χ*^2^ (3) = 3.577, *p* = 0.311
*Psychopathology*																
OCD status, t0 (C-OCS/nC-OCS/no OCS)^a^	90	33/24/ 33	36.7/26.7/ 36.7	45	3/13/ 29	6.7/28.9/ 64.4	28	4/2/22	14.3/7.1/ 78.6	375	9/17/ 349	2.4/4.5/93.1	358	49/56/433	9.1/10.4/ 80.5	*χ*^2^ (6) = 174.335, *p* < 0.001
No psychiatric diagnosis, t0	90	52	57.78	45	30	66.67	28	22	78.57	375	287	57.53	538	391	72.68	*χ*^2^ (3) = 14.178, *p* = 0.003
PHQ-9 total, t0	90	8.18	5.64	45	5.16	4.77	28	4.57	4.30	375	3.73	4.25	538	4.63	4.83	*F*(3, 534) = 23.295, *p* < 0.001, +/+ > −/+, +/−, −/−
OCI-R total score, t0	90	22.77	12.40	45	12.20	7.84	28	10.71	6.85	375	6.42	6.61	538	9.86	10.00	*F*(3, 534) = 103.063, *p* < 0.001 +/+ > −/+, + /−; −/+, + /− > −/−
OCI-R total score, t1	216	30.14	10.25	120	10.67	5.19	71	22.87	6.03	800	6.30	4.59	1207	11.97	11.19	*F*(3, 1203) = 938.860, *p* < 0.001 +/+ > +/− > −/+ > −/−
OCI-R total score, t2	216	30.72	10.29	120	23.19	5.59	71	12.25	4.19	800	6.92	4.84	1207	13.11	11.38	*F*(3, 1203) = 949.629, *p* < 0.001 +/+ > −/+ > +/− > −/−
*Experiential avoidance*																
AAQ-OCD-COVID, t1	215	42.21	18.06	118	25.54	11.36	68	31.56	12.88	787	21.68	9.45	1188	26.34	14.20	*F*(3, 1184) = 173.877, *p* < 0.001 +/+ > +/− > −/+ > −/−
AAQ-OCD-COVID, t2	210	37.80	16.85	117	27.92	13.73	71	22.01	8.85	788	18.85	7.89	1186	23.29	12.93	*F*(3, 1182) = 180.977, *p* < 0.001 +/+ > −/+ > +/− > −/−

^a^C-OCS = contamination-related OCS (OCI-R total score at t0 ≥18 and washing subscale at t0 ≥3); nC-OCS = contamination-unrelated OCS (OCI-R total score at t0 ≥18 and washing subscale at t0 <3); no OCS = OCI-R total score at t0 <18).

^b^Bonferroni-corrected post hoc tests.

*OCS* obsessive-compulsive symptoms*, PHQ-9* Patient Health Questionnaire Depression Module, *OCI-R* Obsessive-Compulsive Inventory-Revised, *AAQ-OCD-COVID* Acceptance and Action Questionnaire for Obsessions and Compulsions (COVID-19 adaption); after adjustment for multiple testing according to the Holm-Bonferroni procedure, only findings with a *p* of .001 or below should be considered statistically significant.

### OCS

When a *t*-test for dependent samples was calculated for the total sample, OCS as measured by the OCI-R total score numerically increased between t1 and t2 (see Table 1), but effects were small, *t*(1206) = 5.220, *p* < 0.001, *d* = 0.15 (CI_95%_ 0.07 to 0.23). Accordingly, the number of participants classified as OCS+ also increased (t1: *n* = 287, 23.8%, t2: *n* = 287, 27.8%, McNemar’s test, df = 1, *p* < 0.001). In addition, we calculated a repeated-measures analysis of variance for the OCI-R total score for the sample with complete data at all three assessment points (*n* = 538). As expected, the OCI-R total score increased over time, *F*(2, 1074) = 21.345, *p* < 0.001, *ƞ*²_part_ = 0.038. On OCI-R subscale level, this increase was also found for washing (*F*(2, 1074) = 134.523, *p* < 0.001, *ƞ*²_part_ = 0.200), obsessing (*F*(2, 1074) = 4.452, *p* = 0.012, *ƞ*²_part_ = 0.008), hoarding (*F*(2, 1074) = 16.261, *p* < 0.001, *ƞ*²_part_ = 0.029), and ordering (*F*(2, 1074) = 20.589, *p* < 0.001, *ƞ*²_part_ = 0.037), but not for the checking (*F*(2, 1074) = 0.493, *p* = 0.611, *ƞ*²_part_ = 0.001), and neutralizing OCI-R subscale (*F*(2, 1074) = 0.570, *p* = 0.563, *ƞ*²_part_ = 0.001).

### OCS trajectory groups (*n* = 1207)

**Fig. 1:**
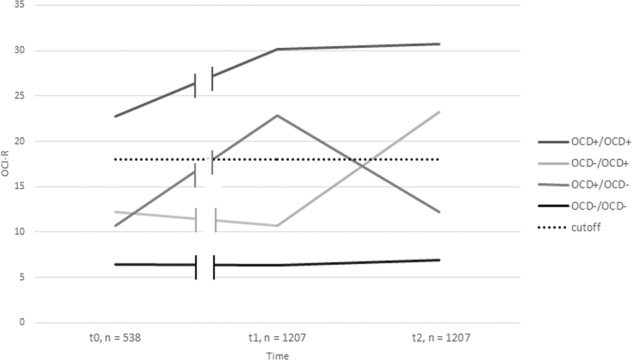
Four trajectories of obsessive-compulsive symptoms (OCS) over the COVID-19 pandemic and OCI-R total score at t0 (March 2014), t1 (March 2020), and t3 (June 2020). The dotted line represents the cut-off point of the German OCI-R, above which symptom severity can be considered clinically relevant.

Regarding the defined trajectory groups using the German OCI-R cut score of 18, 216 people (17.9%) classified as OCS+/OCS+, 71 (5.88%) as OCS+/OCS−, 120 (9.94%) as OCS−/OCS+, and 800 (66.28%) as OCS−/OCS−. Please see Fig. 1 for the mean OCI-R total score of the trajectories groups including t0 and Table 1 for a demographic description of the groups and group comparisons. When trajectory groups were recalculated for the cut score 21, 158 people (13.1%) classified as OCS+/OCS+, 61 (5.1%) as OCS+/ OCS−, 100 (8.3%) as OCS−/OCS+, and 888 (73.6%) as OCS−/OCS−.

### Prediction of trajectory group membership and change over the course of the pandemic

Table 2 shows the hierarchical multinomial regression. In the first step, t0 variables that might have influenced OCS trajectory membership were investigated (i.e., age, sex, education, depression, OCS, contamination relevance of OCS). Male gender was significantly associated with an increased probability of both an OCS+/OCS+ trajectory (*OR* = 1.825, CI_95%_ 1.218 to 2.733) and an OCS+/OCS− trajectory (*OR* = 1.992, CI_95%_ 1.088 to 3.646). Moreover, higher education (A levels) reduced the odds of an OCS+/OCS− trajectory with an odds ratio of 0.388 (CI_95%_ 0.165 to 0.912). Pre-pandemic contamination-related OCS (C-OCS) was significantly associated with increased odds of scoring above the cut-off score one or more of the assessments in 2020, with the highest odds ratios of 31.931 (CI_95%_ 11.923 to 85.514) for the OCS+/OCS+ trajectory (OCS−/OCS+: *OR* = 5.266, CI_95%_ 1.246 to 22.265; OCS+/OCS−: *OR* = 8.176, CI_95%_ 2.112 to 31.644). Pre-pandemic nC-OCS was associated with increased odds of membership in the OCS+/OCS+ trajectory group with an odd ratio of 11.416 (CI_95%_ 4.962 to 26.269) and in the OCS–/OCS+ trajectory group with an odd ratio of 8.836 (CI_95%_ 3.747 to 20.835).

**Table 2:** Association of the factors with group membership (*n* = 1207): hierarchical multinomial regression.

	OCS+/OCS+^b^			OCS−/OCS+^b^			OCS+/OCS−^b^		
Variables	OR	[CI 95%]	*p*	OR	[CI 95%]	*p*	OR	[CI 95%]	*p*
*Block 1*									
Age	0.994	[0.978, 1.011]	0.490	1.005	[0.989, 1.022]	0.528	0.985	[0.964, 1.006]	0.167
Education (A levels)	1.085	[0.606, 1.943]	0.785	1.007	[0.507, 2.000]	0.983	0.388	[0.165, 0.912]	0.030
Sex (1 = f, 2 = m)	1.825	[1.218, 2.733]	0.004	0.925	[0.587, 1.457]	0.735	1.992	[1.088, 3.646]	0.026
PHQ-9, t0	1.066	[0.996, 1.141]	0.067	1.005	[0.935, 1.080]	0.892	1.002	[0.898, 1.119]	0.968
C-OCS, t0^a^	31.931	[11.923, 85.514]	<0.001	5.266	[1.246, 22.265]	0.024	8.176	[2.112, 31.644]	0.002
nC-OCS, t0^a^	11.416	[4.962, 26.269]	<0.001	8.836	[3.747, 20.835]	<0.001	1.905	[0.271, 13.377]	0.517
*Block 2*									
Age	0.998	[0.981, 1.016]	0.850	1.002	[0.985, 1.020]	0.806	0.984	[0.963, 1.006]	0.153
Education (A levels)	0.962	[0.486, 1.904]	0.912	0.998	[0.493, 2.021]	0.996	0.348	[0.144, 0.845]	0.020
Sex (1 = f, 2 = m)	1.746	[1.093, 2.788]	0.020	1.045	[0.652, 1.674]	0.856	2.222	[1.204, 4.102]	0.011
PHQ-9, t0	1.062	[0.989, 1.141]	0.099	1.021	[0.956, 1.090]	0.544	0.995	[0.895, 1.106]	0.925
C-OCS, t0^a^	20.633	[6.495, 65.548]	<0.001	3.185	[0.726, 13.969]	0.125	8.142	[2.093, 31.670]	0.002
nC-OCS, t0^a^	8.962	[3.529, 22.754]	<0.001	7.363	[2.956, 18.340]	<0.001	1.644	[0.231, 11.719]	0.620
AAQ-OCD-COVID, t1	1.145	[1.120, 1.172]	<0.001	1.074	[1.050, 1.098]	<0.001	1.064	[1.035, 1.094]	<0.001
Change in AAQ-OCD-COVID, t1–t2	0.934	[0.911, 0.957]	<0.001	0.922	[0.900, 0.945]	<0.001	1.017	[0.981, 1.055]	0.356

^a^Reference group: no OCS (total score at t0 <18).

^b^Reference group: asymptomatic trajectory (OCS−/OCS−); after adjustment for multiple testing according to the Holm-Bonferroni procedure, only findings with a *p* of .001 or below should be considered statistically significant.

*OCS* obsessive-compulsive symptoms*, OR* Odds ratio, *OCS+/OCS+* continuously symptomatic trajectory with OCS at t1 and t2, *OCS−/OCS+* delayed onset trajectory without OCS at t1 but at t2, *OCS+/OCS−* the recovery group trajectory with OCS at t1 but not at t2, *C-OCS* contamination-related OCS (OCI-R total score at t0 ≥18 and washing subscale at t0 ≥3), *nC-OCS* contamination-unrelated OCS (OCI-R total score at t0 ≥18 and washing subscale at t0 <3), *PHQ-9* the Patient Health Questionnaire Depression Module, *OCI-R* Obsessive-Compulsive Inventory-Revised, *AAQ-OCD-COVID* Acceptance and Action Questionnaire for Obsessions and Compulsions (COVID-19 adaption).

In the second step, when EA at t1 and change from t1 to t2 were added to the model, higher EA at t1 was significantly associated with increased probability of scoring above the cut-off score on one or both of the assessments in 2020, that is, an OCS+/OCS+ trajectory (*OR* = 1.145, CI_95%_ 1.120 to 1.172), and OCS−/OCS+ trajectory (*OR* = 1.074, CI_95%_ 1.050 to 1.098), or an OCS+/OCS− trajectory (*OR* = 1.064, CI_95%_ 1.035 to 1.094). A higher decrease in EA from t1 to t2 significantly reduced the probability of showing an OCS+/OCS+ (*OR* = 0.934, CI_95%_ 0.911 to 0.957) or an OCS−/OCS+ (*OR* = 0.922, CI_95%_ 0.900 to 0.945) trajectory.

The sensitivity analysis calculated for participants with available data at t0, t1, and t2 (*n* = 519) largely confirmed the results (see online supplement Table A). However, the effects of gender were no longer significant, as well as the effects of pre-pandemic contamination-related OCS on the OCS−/OCS+ trajectory. Moreover, a higher level of depression (PHQ-9) at t0 was associated with increased odds of showing an OCS+/OCS+ trajectory with an odds ratio of 1.066 (CI_95%_ 1.002 to 1.1.24). When hierarchical multinomial regressions were recalculated for trajectory groups based on the cut score 21, results remained largely unchanged (see online supplement Table B).

When multiple hierarchical regression models were calculated to predict change in OCS over the pandemic, only age was a statistically significant predictor in the first step (β = −0.09. *p* = 0.044), suggesting that younger age was associated with a higher decrease in OCS. When in the second step EA at t1 and change in EA from t1 to t2 were entered as additional predictors only change in EA was a significant predictor (β = 0.268, *p* < 0.001) suggesting that a decrease in EA predicted the decrease in OCS over the pandemic. The final model was statistically significant (*R* = 0.292, *F* = 5.951, df = 8, 518, *p* < 0.001), explaining 8.5% of the variance.

## Discussion

This study is the first to examine the frequency of different OCS trajectories during the first three months of the COVID-19 pandemic, from March to June 2020, and to investigate factors that may predict OCS development in the general population in Germany. In summary, trajectories over the pandemic largely varied between participants; OCS might occur at various stages of the pandemic and might also occur temporarily. Importantly, and in line with epidemiological data on OCD, e.g., refs. ^1,53^, the majority of the participants scored below the cut-off score for clinically relevant OCS at both assessments (OCS−/OCS−, *n* = 800, 66.3%). Corresponding with previous studies in people with manifest OCD^9–13^ and with a nonclinical sample^15,17,18^, the OCS rate/OCS increased over the course of the pandemic, but only with a small effect size. Of the investigated predictors (gender, age, education, depression, C-OCS, EA), only education, OCS in 2014, and EA at t1 were associated with allocation to the OCS group on one or both of the assessments in 2020.

### Trajectories of OCS during the COVID-19 pandemic

Approximately every third person scored above the cut-off score for clinical OCS in one or more of the assessments in 2020^9,10^. This means that on one or more of the assessments the person scored above the OCI-R cut-off score of 18. Approximately 18% of the participants (*n* = 216) had an OCS+/OCS+ trajectory and scored above the cut-off at both assessments. The two smallest groups were participants with an OCS−/OCS+ trajectory (*n* = 120, 9.9%) or an OCS+/OCS− trajectory (*n* = 71, 5.9%), in which they only scored above the cut-off at t1 or t2, respectively.

Second, of all participants classified as OCS+ at t1 (23.8%, *n* = 287), 75.3% showed an OCS+/OCS+ trajectory, underlining the stability of OCS^31^, and 24.7% (*n* = 71) fell below the German cut-off score at t2. When compared to the general prevalence rate of OCD, the present OCS rate seems high and is much higher than general incidence rates for OCD, which are estimated at 1.2−3.8% (12-month prevalence rate^1,48^). However, one has to keep in mind that OCS status in our study was based on the OCI-R and not on a clinical interview. It fits with a review by Abramowitz et al.^54^ summarizing that “mean percentage of people scoring above the cut-off [of the original OCI-R], weighted by sample size, was 26%” (p. 208). Still, we used the German OCI-R cut-off score of 18, which has been established by Gönner et al.^37^ to differentiate between healthy subjects and patients with OCD and has a sensitivity of 84% and a specificity of 82%. The German cut-off is lower than the cut-off score for the original English version (21 points; sensitivity: 66%; specificity: 64% see Foa et al.^38^). There are pros and cons for various specific cut-off scores. However, when using the OCI-R as a screening tool, a lower cut-off has been recommended to reduce the rate of false negatives^37^. When recalculating the trajectories for the English cut-off of 21 indicating slightly lower rates. Still, 26.5% of the participants (compared to 33.7% when the cut-off 18 was used) scored above the cut-off score one or more of the assessments in 2020, which largely corresponds to findings of an OCD prevalence of 17.93% in Wuhan three months after the quarantine was lifted^16^.

Besides general prevalence rates of OCD, subclinical rates may be considered for comparison. These are quite heterogeneous and rates between 2 and 8% have been reported for Germany^55,56^. In the National Comorbidity Survey Replication in the US, the lifetime rate of experiencing obsessions or compulsions has been estimated at around 28%^1^. However, that study’s methods differ from those of this study (i.e., the choice of diagnostic instruments, the country in which the study was conducted. Thus, the current rates may probably best be compared to the rates in the same online panel. Using the OCI-R as well, 366 (17.9%) of the assessed participants (*N* = 2048) were classified as OCS+ in March 2014, and the mean OCI-R total score was 9.86 (10.00). Because the WHO confirmed that the COVID-19 outbreak was a pandemic about 10 days before t1 (March 11, 2020), a pandemic-related increase in the OCS+ rate (from 17.9% in March 2014 to 23.8% in March 2020) and mean OCS severity (*M* = 11.97, *SD* = 11.19) prior to our 2020 assessment is possible. However, as the time period between the two assessments was so long (6 years) and data for 2014 was only available for a subsample, interpretation of differences in OCS+ rates and OCS severity between 2014 and 2020 is difficult and firm conclusions cannot be drawn.

Third, 120 participants (9.9%) were OCS− at t1 but OCS+ at t2, indicating a delayed onset of OCS during the pandemic. In total, however, only an increase in the OCS+ rate from 24% at t1 to 28% at t2 was found. This corresponds to the increase in mean OCS severity from 11.97 (*SD* = 11.19) from March 2020 to 13.11 (*SD* = 11.38) in June 2020 at a small effect size (*d* = 0.15, CI_95%_ 0.07 to 0.23). This is in line with an increase in OCS reported during other pandemics^57^ and preliminary results of an increase in OCS during the COVID-19 pandemic in clinical^9–13^ and nonclinical samples^15–18^. As in the study by Cox and Olantunji^18^ the effect size for an increase of OCS was small in our study, and the majority of participants were allocated to the OCS– group at both assessments in 2020 (*n* = 800, 66.28% for the cut-off score of 18, *n* = 888, 73.6% for the cut-off score of 21) and did not transition to one of the OCS+ groups.

### Predictors of trajectories and change

We further investigated factors that might predict scoring above the cut-off score one or more of the assessments in 2020. First, the male gender predicted the OCS+/OCS+ and the OCS+/OCS− trajectory with odds ratios between 1.825 and 1.992, meaning that the odds for men to score above the cut-off score on the first assessment in 2020 were almost twice as large as for women. This corresponds to results showing that the male gender is associated with worrying about germs and ritualized cleaning behavior during COVID-19^15^. However, as this result was not confirmed in the sensitivity analyses for complete cases, caution in interpretation is warranted.

Second, higher education reduced the odds of showing an OCS+/OCS− trajectory (with an odds ratio of 0.388). Preliminary findings during the COVID-19 pandemic by Abba-aji et al.^15^, however, reported the opposite for severity OCS. However, as stated above, their study suffers from methodological limitations and ours differed with regard to the methods used (e.g., cross-sectional design, retrospective assessment of pre-pandemic OCS).

Third, contamination-related and contamination-unrelated OCS at t0 was associated with both an OCS+/OCS+ and an OCS−/OCS+ trajectory. Contamination-related OCS at t0 was associated with an OCS+/OCS+ trajectory with an odds ratio of 31.931 (CI_95%_ 11.923 to 85.514), meaning that if a person scored above the OCI-R cut-off for the total score and the cut-off for the washing subscale in 2014, the odds for scoring above the OCI-R cut-off at both assessments in 2020 increased by a factor of 31.9. For the OCS−/OCS+ trajectory the odds ratio was 5.266 (CI_95%_ 1.246 to 22.265) and for the OCS+/OCS− trajectory is was 8.176 (CI_95%_ 2.112 to 31.644). Pre-pandemic contamination-unrelated OCS was also associated with increased odds of scoring above the OCI-R cut-off in 2020, however, only in the OCS+/OCS+ trajectory group with an odd ratio of 11.416 (CI_95%_ 4.962 to 26.269) and the in the OCS–/OCS+ trajectory group with an odd ratio of 8.836 (CI_95%_ 3.747 to 20.835). This finding is only partly in line with prior data collected during the COVID-19 pandemic^9^, which has shown that people with manifest C-OCS (washers) represent a risk group. Due to the stability of OCD^31^, it is understandable that any sort of OCS (both contamination-related and contamination-unrelated) predicts OCS at a later point in time. Still, for contamination-related OCS odds were comparatively high for OCS+/OCS+ trajectory and appeared exclusively for the only OCS+/OCS− trajectory (and not for contamination-unrelated OCS in 2014). However, in the sensitivity analyses calculated for complete cases (supplementary online material Table A) contamination relevance of OCS in 2014 did not predict the OCS−/OCS+ trajectory. Potentially, having previously had C-OCS made people particularly prone to an initial but only short-term OCS response to the pandemic. It seems that the contamination relevance of OCD/OCS is important for the early and immediate development of OCS. This largely corresponds to evidence that contamination/cleaning is one of the OCS symptom dimensions in which the content of concerns fluctuates over time^4^. Future studies with longer assessment periods should further target the question of whether this also applies to symptom severity, as is indicated by the current data.

Finally, we investigated the role of EA as a potential mediator for OCS trajectories: Higher EA at t1 was associated with scoring above the cut-off score one or more of the assessments in 2020 with odds ratios between 1.064 and 1.145. Thus, the reluctance to experience internal states, and in particular COVID-19-related intrusions, at the beginning of the lockdown preceded a less favorable OCS development during the pandemic. Notably, a decrease in EA during the lockdown (from t1 to t2) reduced the probability of showing an OCS+/OCS+ or an OCS−/OCS+ trajectory, with an odds ratio of 0.934 (0.911–0.957) and an odds ratio of 0.922 (0.900–0.945), respectively. These results suggest that EA may mediate the course of OCS. However, this remains to be shown in future studies, as we only investigated (change in) EA as a predictor of the course of OCS. Results were confirmed in the sensitivity analyses and underline the importance of EA as a predictor for psychological disorders (e.g., Kashdan et al.^28^, including the development and maintenance of OCS^26^. So far, the mediating role of EA in OCS has mostly been investigated in cross-sectional designs^24^, as well as over the course of interventions^30^. In our assessment of EA, however, we used an instrument specifically developed for measuring EA in OCS (i.e., the AAQ-OCD^26^) that we further adapted to the COVID-19 pandemic. This was important to measure the change in EA related to COVID-19.

When the change in OCS over the course of the pandemic was predicted, however, only younger age and decrease in EA significantly predicted decrease in OCS; C-OCS in 2014, gender, educational level, depressive symptoms in 2014, and level of EA at the start of the pandemic were not associated with the change in OCS. Differences in results are understandable as the prediction of group membership defined by trajectories of OCS differs from the prediction of overall change in OCS in terms of the assumptions behind these analyses. Although the investigation of trajectory groups assumes that distinct classes in the population exist, the analysis of the metric outcomes assumes that the differences between individuals are quantitative rather than qualitative.

### Limitations

While this study has strengths, such as its longitudinal design, the availability of pre-pandemic data, and its large sample size, it also faces some limitations. First and most importantly, the results are based on a metric self-rating, and cut-off scores were used for diagnostic purposes. This comes with disadvantages, most prominently the loss of information and the ignorance of the statistical uncertainty of measurement. Nevertheless, using categorical rather than metric information focuses on variation at the clinically most important region of the scale, that is, the transition from unproblematic to clinically relevant states (and not, for example, on variation across several unproblematic states). However, using a structured interview would have been optimal for diagnostic purposes as it would have allowed making formal psychiatric diagnoses (e.g., with the Y-BOCS). However, the OCI-R is the most commonly used self-rating instrument for OCD^39^. Moreover, it has been recommended as a screening measure^58^ and has shown good sensitivity and specificity in the German version^44,59^. However, results need to be verified using clinical interviews. Second, given that hoarding is no longer considered a subtype of OCD and that Wootton et al.^60^ have suggested removing the hoarding scale from the OCI-R, our decision to include the hoarding scale may be viewed as a limitation. We decided to include the scale because (1) the hoarding scale was included in the first assessment in 2014 and we wanted to keep the assessment as consistent as possible over time, (2) our analyses were based on dichotomizing using the OCI-R total score, which was established with the hoarding scale included, and (3) an increase in hoarding symptoms has been associated with the COVID-19 pandemic^57^. Third, we only assessed OCS at two-time points during the pandemic. Thus, only linear trajectories were possible to capture and changes in OCS from March-May 2020 could not be captured. Fourth, as compensation for study participation, we offered a self-help manual on self-esteem at t1. While we deem it unlikely that this general-purpose eight-page manual, which was unrelated to the pandemic, substantially changed participants’ OCS, we cannot fully exclude the possibility that it affected trajectories. Fifth, we adapted the AAQ-OCD to COVID-19-related terminology without doing any prior psychometric work. This enabled us to collect data early in the COVID-19 pandemic and, although the internal consistency was acceptable, this does represent a limitation, and further psychometric work is necessary. Sixth, we calculated a large number of statistical tests and not all results withstood correction for multiple testing. Finally, the response rate was low (16%) and responders were older and more likely to be male and to have an A-level degree than nonresponders, limiting the generalizability of the findings.

## Conclusion

In summary, our results show that the majority of participants did not develop clinically relevant OCS during the first months of the pandemic in Germany. Different OCS trajectories were identified, and OCS might occur at various stages of the pandemic and might also occur temporarily. From our data, previous OCS+ status seems to be the most important predictor of classification as OCS+ on one of the two assessments during the pandemic, which leads us to suggest that adhering to pandemic-related measures (e.g., WHO guidelines) does not significantly increase the OCS rate in people who were not previously affected by OCS. The results, however, may point to the sensitization of health care providers regarding the recognition of previous OCS (both contamination-related and contamination-unrelated) and the administration of screening instruments to be followed by interventions to prevent relapse or exacerbation.

Moreover, our results support the hypothesis that EA plays a role in the development and maintenance of OCS and that EA should be considered in illness models of OCD. However, other factors that may influence the course of OCS—risk as well as resilience factors—need to be investigated. For example, intolerance of uncertainty, which has recently been shown to mediate the relationship between COVID-19-related concerns and OCS by Wheaton et al.^25^, may represent a risk factor. In contrast, positive coping styles have been suggested as protective factors^17^. Our results may help to identify people at risk of OCS during a pandemic and to inform models of OCD, as well as interventions targeting OCS.

## Supplementary information

Table A. Sensitivity Analysis for Complete Cases.

Table B. Sensitivity Analysis With OCI-R Cut-off 21 (following Foa et al, 2002).

## Data Availability

Data is available upon request.
