# Supplementary material for: Predictors of trajectories of obsessive-compulsive symptoms during the COVID-19 pandemic in the general population in Germany
Source: Transl Psychiatry. 2021 May 27;11:323. doi: 10.1038/s41398-021-01419-2 (PMC8155650; doi:10.1038/s41398-021-01419-2)
Supplement: Supplementary file 1 — Table A. Sensitivity Analysis for Complete Cases. [file 41398_2021_1419_MOESM1_ESM.docx]

**Table A.** **Sensitivity Analysis for Complete Cases.** Association of the Factors With Group Membership (*n* = 519): Hierarchical Multinomial Regression

|  | OCS+/OCS+ ^b^ | | | | | OCS−/OCS+ ^b^ | | | | | OCS+/OCS− ^b^ | | | | |
| --- | --- | --- | --- | --- | --- | --- | --- | --- | --- | --- | --- | --- | --- | --- | --- |
| Variables | OR | [CI 95%] | | | *p* | OR | [CI 95%] | | | *p* | OR | [CI 95%] | | | *p* |
| *Block 1* |  |  | | |  |  |  | | |  |  |  | | |  |
| Age | 1.003 | [0.981 | , | 1.027] | .766 | 1.021 | [0.993 | , | 1.050] | .137 | 0.982 | [0.950 | , | 1.015] | .283 |
| Education (A levels) | 1.089 | [0.603 | , | 1.966] | .778 | 1.019 | [0.508 | , | 2.044] | .958 | 0.426 | [0.185 | , | 0.981] | .045 |
| Sex (1 = f, 2 = m) | 1.630 | [0.897 | , | 2.995] | .108 | 1.285 | [0.624 | , | 2.645] | .496 | 1.566 | [0.683 | , | 3.591] | .290 |
| PHQ-9, t0 | 1.066 | [1.002 | , | 1.134] | .044 | 1.006 | [0.926 | , | 1.093] | .893 | 0.998 | [0.892 | , | 1.116] | .969 |
| C-OCS, t0 ^a^ | 32.439 | [12.956 | , | 81.219] | <.001 | 3.666 | [0.698 | , | 19.257] | .125 | 6.133 | [1.289 | , | 29.194] | .023 |
| nC-OCS, t0 ^a^ | 10.489 | [4.689 | , | 23.461] | <.001 | 9.668 | [3.794 | , | 24.640] | <.001 | 1.955 | [0.375 | , | 10.194] | .426 |
| *Block 2* |  |  |  |  |  |  |  |  |  |  |  |  |  |  |  |
| Age | 1.009 | [0.982 | , | 1.036] | .528 | 1.028 | [0.998 | , | 1.059] | .066 | 0.979 | [0.946 | , | 1.013] | .216 |
| Education (A levels) | 0.977 | [0.486 | , | 1.961] | .947 | 0.973 | [0.471 | , | 2.011] | .941 | 0.389 | [0.163 | , | 0.928] | .033 |
| Sex (1 = f, 2 = m) | 1.579 | [0.771 | , | 3.231] | .211 | 1.515 | [0.711 | , | 3.225] | .282 | 1.792 | [0.761 | , | 4.218] | .182 |
| PHQ-9, t0 | 1.064 | [0.990 | , | 1.144] | .091 | 1.02 | [0.94 | , | 1.108] | .632 | 0.984 | [0.875 | , | 1.106] | .784 |
| C-OCS, t0 ^a^ | 25.789 | [8.709 | , | 76.363] | <.001 | 1.874 | [0.321 | , | 10.928] | .485 | 7.056 | [1.388 | , | 35.884] | .019 |
| nC-OCS, t0 ^a^ | 8.779 | [3.329 | , | 23.154] | <.001 | 8.554 | [3.154 | , | 23.202] | <.001 | 1.865 | [0.347 | , | 10.017] | .468 |
| AAQ-OCD-COVID, t1 | 1.154 | [1.116 | , | 1.193] | <.001 | 1.060 | [1.021 | , | 1.101] | .002 | 1.066 | [1.019 | , | 1.114] | .005 |
| Change in AAQ-OCD-COVID, t1–t2 | 0.941 | [0.908 | , | 0.975] | .001 | 0.904 | [0.869 | , | 0.939] | <.001 | 1.010 | [0.959 | , | 1.065] | .699 |

Notes: ^a^ reference group: no OCS (total score at t0 < 18); ^b^ reference group: asymptomatic trajectory (OCS-/OCS-); abbreviations: OR = Odds ratio; OCS+/OCS+ = continuously symptomatic trajectory with OCS at t1 and t2; OCS-/OCS+ = delayed onset trajectory without OCS at t1 but at t2; OCS+/OCS- = the recovery group trajectory with OCS at t1 but not at t2; C-OCS = contamination-related OCS (OCI-R total score at t0 ≥ 18, and washing subscale at t0 ≥ 3); nC-OCS = contamination-unrelated OCS (OCI-R total score at t0 ≥ 18, and washing subscale at t0 < 3); PHQ-9 = the Patient Health Questionnaire Depression Module; OCI-R = Obsessive-Compulsive Inventory-Revised; AAQ-OCD-COVID = Acceptance and Action Questionnaire for Obsessions and Compulsions (COVID-19 adaption).
