# Supplementary material for: Predictors of trajectories of obsessive-compulsive symptoms during the COVID-19 pandemic in the general population in Germany
Source: Transl Psychiatry. 2021 May 27;11:323. doi: 10.1038/s41398-021-01419-2 (PMC8155650; doi:10.1038/s41398-021-01419-2)
Supplement: Supplementary file 2 — Table B. Sensitivity Analysis With OCI-R Cut-off 21 (following Foa et al, 2002). [file 41398_2021_1419_MOESM2_ESM.docx]

**Table B. Sensitivity Analysis With OCI-R Cut-off 21 (following Foa et al, 2002).** Association of the Investigated Factors With Group Membership (*n* = 1207): Hierarchical Multinomial Regression

|  | OCS+/OCS+ ^b^ | | | | | OCS-/OCS+ ^b^ | | | | | OCS+/OCS- ^b^ | | | | |
| --- | --- | --- | --- | --- | --- | --- | --- | --- | --- | --- | --- | --- | --- | --- | --- |
| Variables | OR | [CI 95%] | | | *p* | OR | [CI 95%] | | | *p* | OR | [CI 95%] | | | *p* |
| *Block 1* |  |  | | |  |  |  | | |  |  |  | | |  |
| Age | 0.986 | [0.967 | , | 1.004] | .130 | 1.002 | [0.983 | , | 1.022] | .815 | 1.004 | [0.982 | , | 1.026] | .728 |
| Education (A-level) | 0.894 | [0.442 | , | 1.808] | .756 | 0.880 | [0.435 | , | 1.780] | .721 | 0.831 | [0.344 | , | 2.008] | .680 |
| Sex | 2.169 | [1.351 | , | 3.481] | .001 | 1.290 | [0.791 | , | 2.106] | .308 | 1.802 | [0.980 | , | 3.315] | .058 |
| PHQ-9, t0 | 1.060 | [0.983 | , | 1.144] | .129 | 1.022 | [0.949 | , | 1.099] | .567 | 1.046 | [0.961 | , | 1.139] | .300 |
| C-OCS, t0 ^a^ | 73.061 | [23.715 | , | 225.086] | <.001 | 11.719 | [2.969 | , | 46.255] | <.001 | 12.485 | [3.113 | , | 50.071] | <.001 |
| nC-OCS, t0 ^a^ | 17.488 | [6.606 | , | 46.299] | <.001 | 6.105 | [2.044 | , | 18.233] | .001 | 1.193 | [0.146 | , | 9.766] | .870 |
| *Block 2* |  |  |  |  |  |  |  |  |  |  |  |  |  |  |  |
| Age | 0.988 | [0.968 | , | 1.008] | .243 | 0.998 | [0.978 | , | 1.018] | .818 | 1.004 | [0.982 | , | 1.027] | .704 |
| Education (A-level) | 0.726 | [0.313 | , | 1.684] | .456 | 0.919 | [0.451 | , | 1.869] | .815 | 0.799 | [0.319 | , | 1.997] | .630 |
| Sex | 1.981 | [1.139 | , | 3.444] | .015 | 1.398 | [0.856 | , | 2.283] | .181 | 1.931 | [1.025 | , | 3.638] | .042 |
| PHQ-9, t0 | 1.040 | [0.959 | , | 1.128] | .338 | 1.016 | [0.951 | , | 1.086] | .630 | 1.018 | [0.930 | , | 1.113] | .699 |
| C-OCS, t0 ^a^ | 47.927 | [12.046 | , | 190.686] | <.001 | 7.815 | [1.955 | , | 31.229] | .004 | 11.253 | [2.749 | , | 46.066] | .001 |
| nC-OCS, t0 ^a^ | 18.264 | [6.136 | , | 54.361] | <.001 | 6.382 | [2.052 | , | 19.852] | .001 | 1.287 | [0.178 | , | 9.306] | .803 |
| AAQ-OCD-COVID, t1 | 1.150 | [1.121 | , | 1.179] | <.001 | 1.058 | [1.035 | , | 1.082] | <.001 | 1.091 | [1.064 | , | 1.119] | <.001 |
| Change in AAQ-OCD-COVID, t1-t2 | 0.933 | [0.909 | , | 0.958] | <.001 | 0.935 | [0.914 | , | 0.957] | <.001 | 1.001 | [0.967 | , | 1.036] | .970 |

Notes: ^a^ reference group: no OCS (total score at t0 < 21); ^b^ reference group: asymptomatic trajectory (OCS-/ OCS-); abbreviations: OCS+/ OCS+ = continuously symptomatic trajectory with OCS at t1 and t2; OCS -/ OCS+ = delayed onset trajectory without OCS at t1 but at t2; OCS+/ OCS- = the recovery group trajectory with OCS at t1 but not at t2; C-OCS = contamination-related OCS (OCI-R total score at t0 ≥ 21, and washing subscale at t0 ≥ 3); nC-OCS = contamination-unrelated OCS (OCI-R total score at t0 ≥ 21, and washing subscale at t0 < 3); PHQ-9 = the Patient Health Questionnaire Depression Module; OCI-R = Obsessive-Compulsive Inventory-Revised; AAQ-OCD-COVID = Acceptance and Action Questionnaire for Obsessions and Compulsions (COVID-19 adaption).
